# Resveratrol promotes expression of SIRT1 and StAR in rat ovarian granulosa cells: an implicative role of SIRT1 in the ovary

**DOI:** 10.1186/1477-7827-10-14

**Published:** 2012-02-23

**Authors:** Yoshihiro Morita, Osamu Wada-Hiraike, Tetsu Yano, Akira Shirane, Mana Hirano, Haruko Hiraike, Satoshi Koyama, Hajime Oishi, Osamu Yoshino, Yuichiro Miyamoto, Kenbun Sone, Katsutoshi Oda, Shunsuke Nakagawa, Kazuyoshi Tsutsui, Yuji Taketani

**Affiliations:** 1 Department of Obstetrics and Gynecology, Graduate School of Medicine, The University of Tokyo, 7-3-1, Hongo, Bunkyo-ku, Tokyo 113-8655, Japan; 2 Department of Obstetrics and Gynecology, School of Medicine, Teikyo University, 2-11-1 Kaga, Itabashi-ku, Tokyo 173-8605, Japan; 3 Laboratory of Integrative Brain Sciences, Department of Biology, Waseda University, 2-2, Wakamatsuchou, Shinjuku-ku, Tokyo, 162-8480, Japan

**Keywords:** SIRT1, Resveratrol, Ovary, Granulosa cells, Luteinization

## Abstract

**Background:**

Resveratrol is a natural polyphenolic compound known for its beneficial effects on energy homeostasis, and it also has multiple properties, including anti-oxidant, anti-inflammatory, and anti-tumor activities. Recently, silent information regulator genes (Sirtuins) have been identified as targets of resveratrol. Sirtuin 1 (SIRT1), originally found as an NAD^+^-dependent histone deacetylase, is a principal modulator of pathways downstream of calorie restriction, and the activation of SIRT1 ameliorates glucose homeostasis and insulin sensitivity. To date, the presence and physiological role of SIRT1 in the ovary are not known. Here we found that SIRT1 was localized in granulosa cells of the human ovary.

**Methods:**

The physiological roles of resveratrol and SIRT1 in the ovary were analyzed. Immunohistochemistry was performed to localize the SIRT1 expression. SIRT1 protein expression of cultured cells and luteinized human granulosa cells was investigated by Western blot. Rat granulosa cells were obtained from diethylstilbestrol treated rats. The cells were treated with increasing doses of resveratrol, and subsequently harvested to determine mRNA levels and protein levels. Cell viability was tested by MTS assay. Cellular apoptosis was analyzed by caspase 3/7 activity test and Hoechst 33342 staining.

**Results:**

SIRT1 protein was expressed in the human ovarian tissues and human luteinized granulosa cells. We demonstrated that resveratrol exhibited a potent concentration-dependent inhibition of rat granulosa cells viability. However, resveratrol-induced inhibition of rat granulosa cells viability is independent of apoptosis signal. Resveratrol increased mRNA levels of SIRT1, LH receptor, StAR, and P450 aromatase, while mRNA levels of FSH receptor remained unchanged. Western blot analysis was consistent with the results of quantitative real-time RT-PCR assay. In addition, progesterone secretion was induced by the treatment of resveratrol.

**Conclusions:**

These results suggest a novel mechanism that resveratrol could enhance progesterone secretion and expression of luteinization-related genes in the ovary, and thus provide important implications to understand the mechanism of luteal phase deficiency.

## Background

The study of natural compounds with pharmacological activity has become an emerging trend in nutritional and pharmacologic research. Polyphenols represent a vast group of compounds having aromatic ring, characterized by the presence of one or more hydroxyl groups with various structural complexities. Resveratrol (trans-3, 5, 40-trihydroxystilbene) is a natural polyphenol synthesized by plants as a phytoalexin that becomes activated under stress conditions such as ultraviolet radiation and fungal infection [1,2]. It can be found in berries, nuts and some medicinal plants, and mainly present in the skin of grapes and thus in red wine [3]. Previous studies have reported its anti-oxidant, anti-inflammatory, and growth-inhibitory activities using several cancer cell lines and animal models [2,4,5]. These properties of resveratrol have been linked to the inhibition of proliferation in association with cell cycle arrest and apoptotic cell death typically observed in vitro at concentrations in the range of 10-300 μM [5-7]. Thus, resveratrol has activity in regulating multiple cellular events associated with carcinogenesis, and the activation of SIRT1 is postulated to be a key event to elucidate the pathophysiology of resveratrol [8,9]. SIRT1, the mammalian homologue of yeast Sir2 (silent information regulator 2), functions as an NAD^+^-dependent class III histone deacetylase. SIRT1 deacetylates multiple targets in mammalian cells, including p53, FOXO1, FOXO3, PGC-1α, liver X receptor, NBS1 and hypoxia-inducible factor 2α [10,11]. By regulating these molecules, SIRT1 functions as a master regulator of energy homeostasis, gene silencing, metabolism, genomic stability, and cell survival.

The ability of the ovary to produce growing follicles that ovulate is the basis of female fertilization. A critical feature of ovarian granulosa cell (GC) function is the differentiation of the ovulatory follicle into the corpus luteum which mainly produces progesterone (P4) that is important for the maintenance of pregnancy. Recently, it has been suggested that SIRT1 activator resveratrol plays a role in reproductive biology. Resveratrol was shown to modulate theca cell proliferation [12], and methylated resveratrol analogues possessed biological activities in swine GCs [13]. In the present study, to assess a role of SIRT1 in the regulation of reproductive axis in female, we investigated the expression of endogenous SIRT1 in human and rat GCs and the effect of resveratrol on cellular viability and steroidogenesis in rat GCs.

## Methods

### Chemicals

Diethylstilbestrol (DES) and resveratrol were purchased from Sigma-Aldrich (St. Louis, MO, USA). All other chemicals, unless otherwise mentioned, were obtained from Sigma-Aldrich.

### Human cancer cell lines and primary human GCs

Human cervical cancer cell line HeLa was purchased from American Type Culture Collection (Manassas, VA, USA). Human ovarian granulosa-like tumor cell line KGN, which originated from a Stage III granulosa cell carcinoma in a 63-year-old Japanese women [14], was obtained from RIKEN Cell Bank of Japan (Tsukuba, Japan). Primary human GCs were obtained from patients undergoing transvaginal oocyte retrieval for in vitro fertilization at the University of Tokyo Hospital. The method to purify human GCs was described previously [15]. The study was approved by the Institutional Review Board of the University of Tokyo, and written informed consent for the research use of human GCs was obtained from each patient. These cells were maintained in Dulbecco's modified Eagle Medium (DMEM)/F12 medium (Invitrogen, Carlsbad, CA, USA) supplemented with 10% charcoal-stripped fetal bovine serum (FBS; Invitrogen), 100 U/ml penicillin, 100 μg/ml streptomycin and 0.25 μg/ml amphotericin B in a humidified atmosphere of 5% CO_2 _and 95% air at 37°C.

### Preparation and culture of rat GCs

Guidelines for the care and use of laboratory animals as adopted and promulgated by the University of Tokyo were followed. Twenty-three-day-old immature female Wistar rats were purchased from CLEA Japan, Inc. (Tokyo, Japan) and housed in a temperature-controlled room with a 12 h light/12 h dark schedule. Pelleted food and water were provided ad libitum. Rats were implanted with SILASTIC capsules (Dow Corning, Corp., Midland, MI, USA) containing 10 mg DES to increase GC number [16], and killed 4 days later by cervical dislocation. Removed ovaries were immediately cleaned of surrounding connective tissues and placed into DMEM/F12 medium supplemented with 10% charcoal-stripped FBS, 100 U/ml penicillin, 100 μg/ml streptomycin and 0.25 μg/ml amphotericin B. GCs were harvested by needle puncture of ovarian follicles, suspended in the medium, and purified by filtration with a 100-μm cell strainer and then a 40-μm cell strainer (BD Biosciences, Bedford, MA, USA). Isolated GCs were washed twice by centrifugation at 200 × g for 5 min and cultured in the medium in a humidified atmosphere of 5% CO_2 _and 95% air at 37°C [17].

### Tissue samples and immunohistochemistry

The ovarian tissues used in this study were obtained from 5 female patients with regular menstrual cycles who were taking no hormonal drugs and underwent radical or extended hysterectomy for carcinoma of the uterine cervix and endometrium. The female patients were 32-44 years old at the time of operation and the operations were performed in proliferative phase of the menstrual cycle. The study was approved by the Institutional Review Board of the University of Tokyo, and written informed consent was obtained in each instance. Immunohistochemistry was performed as described previously [18]. Paraffin sections (4 μm) were dewaxed in xylene and rehydrated through graded ethanol to water. Antigens were retrieved by boiling in 10 mM citrate buffer (pH 6.0) for 30 min. The cooled sections were incubated in DAKO REAL Peroxidase-Blocking solution (DAKO, Carpinteria, CA, USA) for 30 min to quench endogenous peroxidase. To block the nonspecific binding, sections were incubated in PBS containing 3% BSA and 0.5% Nonidet P-40 for 10 min at room temperature. Sections were then incubated with the rabbit polyclonal antibody to SIRT1 (sc-15404, Santa Cruz Biotechnology, Inc., Santa Cruz, CA, USA) in DAKO REAL Antibody Diluent (DAKO) overnight at 4°C. Negative controls were incubated with preimmune serum IgG fraction. ChemMate EnVision Detection system (DAKO) was used to visualize the signal. The sections were developed with 3,3'-diaminobenzidine tetrahydrochloride substrate (DAKO), lightly counterstained with ae's hematoxylin (Wako Chemical, Tokyo, Japan), dehydrated through ethanol series and xylene, and mounted.

### Western blotting

HeLa cells, KGN cells, and human GCs were seeded into 6-cm culture dishes (BD Biosciences) at a density of 1 × 10^6 ^cells/dish in 3 ml of the culture medium. After 48 h, the cells were harvested with trypsin (0.05%)/EDTA (0.02%) and scraped into the lysis buffer containing 50 mM Tris-HCl (pH 8.0), 150 mM NaCl, 0.02% sodium azide, 0.1% sodium dodecyl sulfate, 1% Nonidet P-40, and 0.5% sodium deoxycholate for 30 min on ice. Rat GCs were seeded into 10-cm culture dishes (BD Biosciences) at a density of 2-3 × 10^6 ^cells/dish in 10 ml of the culture medium. After 48 h, the medium was replaced with fresh medium containing 1% charcoal-stripped FBS and 100 μM of resveratrol, and cell culture was continued. Thereafter GCs were harvested, and lysed. Insoluble material was removed by centrifugation at 12,000 × g, for 20 min at 4°C. The supernatants were recovered, and the protein concentrations were measured using Bio-Rad protein assay reagent (Bio-Rad Lab., Hercules, CA, USA). Equivalent amounts of lysate protein (30 μg were subjected to 10% SDS-PAGE and electrophoretically transferred onto polyvinylidene difluoride membranes (Millipore Corp., Billerica, MA, USA). After blocking nonspecific binding sites by incubation for 1 h with Tris-buffered saline (25 mM Tris and 150 mM NaCl, pH 7.6) containing 5% nonfat milk and 0.2% Tween 20, the membranes were blotted with the primary antibodies overnight at 4°C. The primary antibodies used were anti-DBC1 [19] and anti-P450 aromatase (P450arom; MCA2077S, AbD serotec, Oxford, UK). Anti-SIRT1 (sc-15404), anti-StAR (sc-25806), and anti-LH receptor (LH-R; sc-25828) were purchased from Santa Cruz Biotechnology Inc. (Santa Cruz). Reactive proteins were detected with horseradish peroxidase-conjugated secondary antibodies (Cell Signaling Technology, Inc., Beverly, MA, USA) for 60 min at room temperature and developed with ECL Plus western blotting detection reagents (GE Healthcare, Little Chalfont, UK). The membranes were stripped with the buffer containing 100 mM 2-mercaptoethanol, 2% SDS and 62.5 mM Tris- HCl (pH 6.7), then reprobed with mouse monoclonal antibody to β-Actin (sc-47778, Santa Cruz Biotechnology, Inc.) to confirm equivalent protein loading. The images were scanned by the luminescent image analyzer Image Quant LAS 4000 mini (GE Healthcare).

### Granulosa cell progesterone production

Culture media for the Western blot were collected, frozen, and stored at -20°C until P4 determination by Progesterone EIA kit (Cayman Chemical Co., Ann Arbor, MI, USA). P4 assay was performed according to the manufacture's instruction. The data are expressed as the amount of steroids (pg/ml) secreted. The results are representative of three to four independent cultures with each condition in quadruplet.

### Cell viability assay

Viability of rat GCs was examined by using the 3-(4,5-dimethylthiazol-2-yl)-5- (3-carboxymethoxyphenyl)-2-(4-sulfophenyl)-2H-tetrazolium, inner salt (MTS) assay kit (CellTiter 96 Aqueous One Solution Cell Proliferation Assay; Promega, Madison, WI, USA) according to the manufacturer's instructions. Briefly, cells were seeded into 96-well plates (BD Biosciences) at a density of 1 × 10^4 ^cells/well in 100 μL of the culture medium. After 48 h, the medium was replaced with fresh medium containing 1% charcoal-stripped FBS and various concentrations of resveratrol, and cell culture was continued for a further 72 h. Resveratrol was dissolved in dimethyl sulfoxide and diluted with the medium to yield desired concentrations. The final concentration of dimethyl sulfoxide never exceeded 0.05%. The effects of resveratrol were investigated at concentrations between 10 and 100 μM in consideration of those used in the other studies where resveratrol inhibited the proliferation of various cell types at concentrations in the range of 10-300 μM [5-7]. Finally, the medium was replaced with 100 μL of fresh medium containing 20 μL of MTS solution and incubated for an additional 4 h. Mitochondrial dehydrogenase enzymes of viable cells converted MTS tetrazolium into a colored formazan product. The optical density of samples was read at 490 nm in the spectrophotometric microplate reader (BioTek, Winooski, VT, USA).

### Reverse transcription and quantitative real-time PCR

Rat GCs were seeded into 6-cm culture dishes (BD Biosciences) at a density of 1 × 10^6 ^cells/dish in 3 ml of the culture medium. After 48 h, the medium was replaced with fresh medium containing 1% charcoal-stripped FBS and various concentrations of resveratrol (10-100 μM, and cell culture was continued for a further 24 h. Total cellular RNA was extracted using RNeasy Mini Kit (Qiagen, Hilden, Germany) and quantified by measuring absorbance at 260 nm and stored at -80° until assay. The mRNA levels of relevant molecules were measured by quantitative real-time RT-PCR using One Step SYBR PrimeScript RT-PCR Kit (TaKaRa Bio. Inc., Tokyo, Japan) in the Light Cycler (Roche Applied Science, Mannheim, Germany). Accumulated levels of fluorescence were analyzed by the second-derivative method after the melting-curve analysis, and then the expression levels of target genes were normalized to the expression level of β-Actin in each sample. Primer pairs of analyzed mRNA are described in Table 1.

**Table 1:** Primer sequences used for quantitative real-time PCR

*Gene*	Primers	Primer Sequence	Expected size in base pair
*β-actin*	Sense	CGAGTACAACCTTCTTGCAG	207
	Antisense	TTCTGACCCATACCCACCAT	
*Bax*	Sense	GAATTGGCGATGAACTGGAC	157
	Antisense	GCAAAGTAGAAAAGGGCAACC	
*Bcl2*	Sense	AACATCGCTCTGTGGATGAC	150
	Antisense	GAGCAGCGTCTTCAGAGACA	
*DBC1*	Sense	TCTCCAAGTCTCGCCTGTG	158
	Antisense	CTCTGTTGCCTCCAACCAGT	
*FSH-R*	Sense	ATGGCCCCCATTTCATTCTT	82
	Antisense	ACTAGGAGAATCTTGGCCTTGGA	
*LH-R*	Sense	ATTGACACTCTGCTTAACTTTCCATCT	82
	Antisense	TGGCCATGAGGTACTCATGATCT	
*p450arom*	Sense	TCCTCAGCAGAGAAACTGGAAGA	151
	Antisense	CGTACAGAGTGACGGACATGGT	
*SIRT1*	Sense	TGTTTCCTGTGGGATACCTGA	137
	Antisense	TGAAGAATGGTCTTGGGTCTTT	
*StAR*	Sense	AGGAAAACAGAACTGAGGCTTAGAATA	93
	Antisense	AAGGTTTCATAGATACCTGTCCCTTAAC	

### Caspase-3/7 activity assay

Apoptosis executioner caspase-3/7 activity in rat GCs was measured using the Apo-ONE Homogeneous Caspase-3/7 Assay kit (Promega) according to the manufacturer's instructions. Briefly, cells were seeded into 96-well plates (BD Biosciences) at a density of 1 × 10^4 ^cells/well in 100 μL of the culture medium. After 48 h, the medium was replaced with fresh medium containing 1% charcoal-stripped FBS and various concentrations of resveratrol (10- 100 μM), and cell culture was continued for 6, 12 and 24 h. Caspase-3/7 activity was measured at excitation wavelength 485 nm and emission wavelength 528 nm in the spectrophotometric microplate reader (BioTek).

### Hoechst 33342 nuclear staining

Hoechst staining was performed to confirm the apoptotic profile as a result of morphological change in the nucleus in which Hoechst 33342 binds specifically to A-T base region in DNA and emits fluorescence. Rat GCs were seeded into 8-well chamber slides (Nalge Nunc International, Naperville, IL, USA) at a density of 1 × 10^5 ^cells/well in 400 μl of the culture medium. After 48 h, the medium was replaced with fresh medium containing 1% charcoal-stripped FBS and resveratrol (50 and 100 μM), and cell culture was continued for 6, 12, 24 and 48 h. Finally, cells were rinsed in PBS and fixed with 4% paraformaldehyde in PBS (pH 7.4) at room temperature for 30 min. Then cells were rinsed in PBS twice and stained with Hoechst 33342 (10 μg/ml in PBS) for 3 min. The specimens were mounted with Vectashield medium (Vector Labs. Inc., Burlingame, CA, USA) and photographs were taken at X200 magnification under a fluorescent confocal microscope (Carl-Zeiss MicroImaging Inc., Oberkochen, Germany).

### Statistical analysis

Data represent the mean ± SEM from at least three independent experiments. Statistical analyses were carried out by one-way ANOVA with *post-hoc *test for multiple comparisons by using StatView software (SAS Institute Inc., Cary, NC, USA). *P *< 0.05 was considered statistically significant.

## Results

### Expression of SIRT1 protein in human GCs

**Figure 1:**
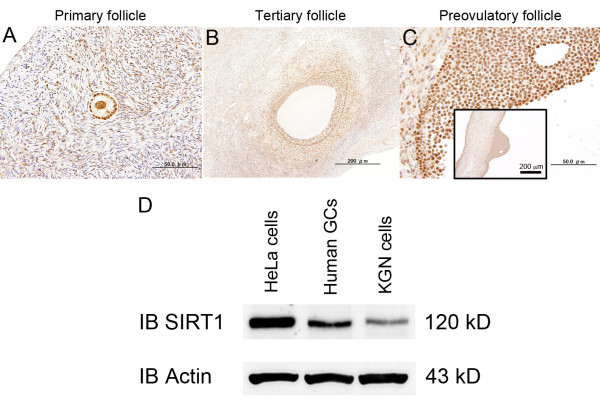
**Expression of SIRT1 protein in human GCs**. Immunohistochemical detection of SIRT1 in the human ovary (A, primary follicle; B, tertiary follicle; C, preovulatory follicle). Representative data from five specimens were shown. Nuclei of GCs were positively stained with anti-SIRT1 antibody at various stages of follicular development. Bars indicate (A) 50 μm, (B) 200 μm, and (C) 50 μm in high-power field and 200 μm in low-power field. SIRT1 was detected in primary (A), tertiary (B), and preovulatory (C) follicles of granulosa cells. Note that a part of the theca cells and oocyte nucleus are stained. Negative controls included a section incubated with preimmune rabbit IgG. (D) Western blotting revealed the presence of SIRT1 in KGN cells, human GCs, and HeLa cells (positive control).

We investigated the localization of SIRT1 protein in the human ovary using immunohistochemistry. Expression of SIRT1 was observed in nuclei of GCs at various stages of follicular development (Figure 1A-C). Part of the theca interstitial cells and the oocyte were also found to have positive signals. To confirm the expression of SIRT1, luteinized human granulosa cells were obtained from women undergoing in vitro fertilization program, and Western blot analysis revealed the expression of SIRT1 protein in KGN cells and human GCs (Figure 1D). HeLa cells were used as a positive control for Western blot because we [19] and other investigators [20] have detected the expression of SIRT1 protein.

### Effect of resveratrol on cell viability and expression of SIRT1 and DBC1 in cultured rat GCs

**Figure 2:**
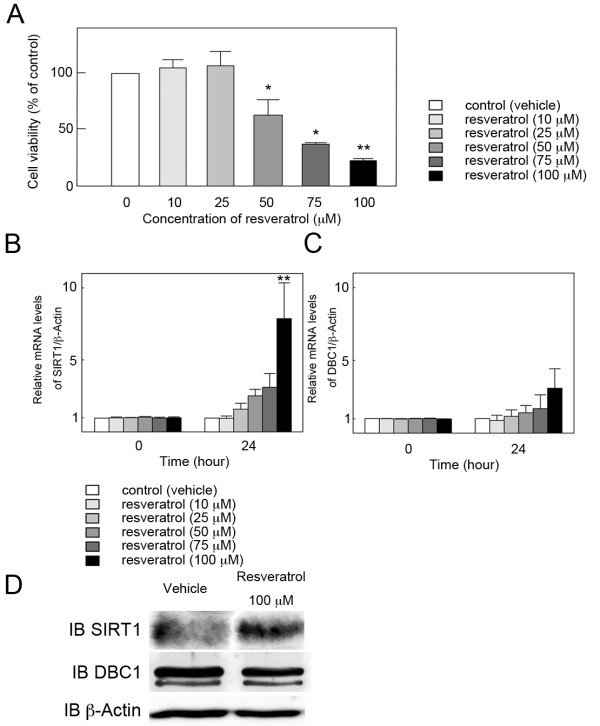
**Effect of resveratrol on cell viability and expression of SIRT1 and DBC1 in cultured rat GCs**. (A) Effect of resveratrol on cell viability at 72 h was estimated by MTS assay. Results are shown as the mean percentage of the untreated control ± SEM (bars) of eight wells of three independent experiments * *p *< 0.05 *vs*. control. ** *p *< 0.01 *vs*. control. (B and C) Effect of resveratrol on mRNA levels of (B) SIRT1 and (C) DBC1 was investigated by quantitative real-time RT-PCR. The mRNA level of the untreated control was arbitrarily set at 1.0, and that of the treatment group was estimated relative to the control value. Results are shown as the mean ± SEM (bars) of three independent experiments. ** *p *< 0.01 *vs*. control. (D) Effect of resveratrol on protein levels of SIRT1 was investigated by Western blot. Resveratrol treatment resulted in an increased expression of SIRT1 protein, and the results were consistent with that of quantitative real time RT-PCR. Three independent experiments were performed and a representative result is shown.

To determine whether activation of SIRT1 by resveratrol affects rat GC viability, the extent of cell viability was measured by MTS assay. Resveratrol, at concentrations between 50 and 100 μM, produced a dose-dependent inhibition of cell viability after 72 h of treatment, with the maximal effect (reduction to 22.8 ± 4.4% of the control) being observed at 100 μM (Figure 2A). Recent studies have shown that DBC1 promotes p53-mediated apoptosis through specific inhibition of deacetylase activity of SIRT1 [21,22]. To determine whether the inhibitory effect on cell viability by resveratrol is related to the change in SIRT1 activation, the effect of resveratrol on mRNA levels of SIRT1 and DBC1, a negative regulator of SIRT1, was investigated by quantitative real-time RT-PCR in cultured rat GCs. After 24 h culture of rat GCs, mRNA levels of SIRT1 significantly increased at 100 μM (Figure 2B), while those of DBC1 remained unchanged (Figure 2C). Western blot analysis was also performed to confirm the result of quantitative real time RT-PCR and resveratrol-dependent induction of SIRT1 protein was observed (Figure 2D)

### Effect of resveratrol on cell-death machinery in cultured rat GCs

**Figure 3:**
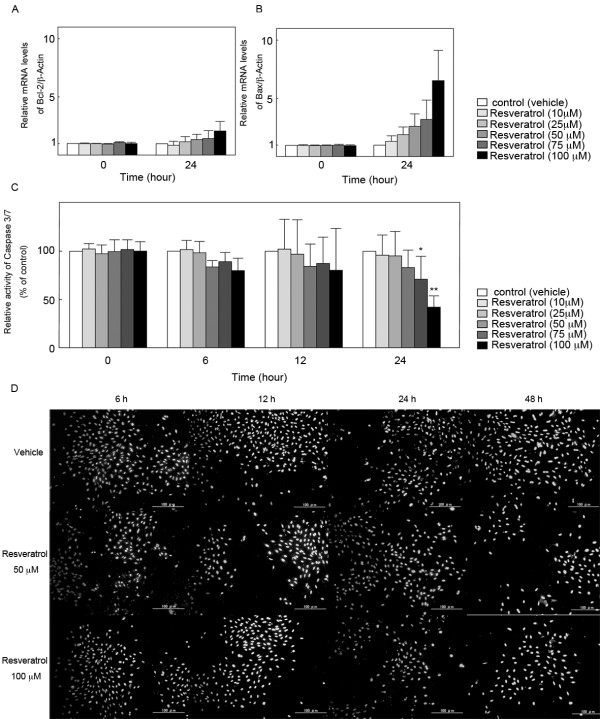
**Effect of resveratrol on cell-death machinery in cultured rat GCs**. Effect of resveratrol on mRNA levels of (A) Bcl-2 and (B) Bax was investigated by quantitative real-time RT-PCR. The mRNA level of the untreated control was arbitrarily set at 1.0, and that of the treatment group was estimated relative to the control value. Results are shown as the mean ± SEM (bars) of three independent experiments. (C) Caspase-3/7 activity was measured by the Apo-ONE Homogeneous Caspase-3/7 Assay kit at 6, 12 and 24 h. Results are shown as the mean percentage of the untreated control ± SEM (bars) of eight wells of three independent experiments. (D) Hoechst 33342 staining of resveratrol-treated rat GCs at 6, 12, 24, and 48 h. * *p *< 0.05 *vs*. control. ** *p *< 0.01 *vs*. control.

Resveratrol has been shown to induce cell-cycle arrest and apoptosis in various cell lines [5,12]. To determine whether the reduction of the viability of rat GCs by resveratrol is due to the induction of apoptosis, the effect of resveratrol on mRNA levels of the representative apoptosis promoter Bax and inhibitor Bcl-2 was analyzed by quantitative real-time RT-PCR in cultured rat GCs at concentrations of resveratrol between 10 and 100 μM. The significant change in mRNA levels of Bax and Bcl-2 was not found at 24 h (Figure 3A, B). Apoptosis executioner caspase-3/7 activity was measured in cultured rat GCs at concentrations of resveratrol ranging from 10 to 100 μM and at various time points (6, 12, and 24 h). Resveratrol significantly inhibited caspase-3/7 activity at 75 and 100 μM after 24 h of treatment (Figure 3C). Furthermore, the effect of resveratrol on the incidence of apoptotic cells was investigated by Hoechst 33342 nuclear staining. Resveratrol (50 and 100 μM) showed no typical apoptotic changes including nuclear shrinkage, chromatin condensation, and nuclear fragmentation in cultured rat GCs at 6, 12, 24, and 48 h (Figure 3D).

### Effect of resveratrol on folliculogenesis-related molecules in cultured rat GCs

**Figure 4:**
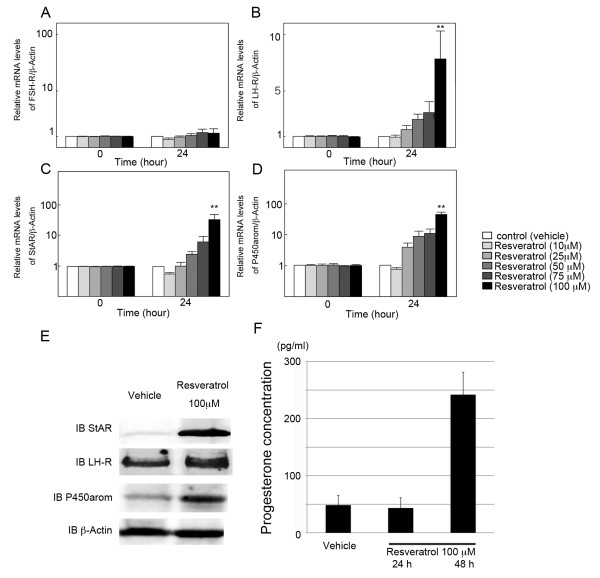
**Effect of resveratrol on folliculogenesis-related molecules in cultured rat GCs**. (A-D) Effect of resveratrol on mRNA levels of (A) FSH-R, (B) LH-R, (C) StAR and (D) P450arom was investigated by quantitative real-time RT-PCR. The mRNA level of the untreated control was arbitrarily set at 1.0, and that of the treatment group was estimated relative to the control value. Results are shown as the mean ± SEM (bars) of three independent experiments. ** *p *< 0.01 *vs*. control. (E) Effect of resveratrol on protein levels of StAR and P450arom was investigated by Western blot. Resveratrol treatment resulted in an increased expression of StAR, LH-R, and P450arom, and the results were consistent with those of quantitative real time RT-PCR. Three independent experiments were performed and a representative result is shown. (F) Effect of resveratrol on P4 secretion by rat granulosa cells. P4 secretion was measured by EIA protocol in culture medium of granulosa cells after 24 to 48 h of culture in DMEM/F-11 medium in the presence of resveratrol (100 μM). The data are expressed as the amount of steroids (pg/ml) secreted. The results, expressed as means ± SEM, are representative of three to four independent cultures with each condition in quadruplet.

The Effect of resveratrol on mRNA levels of folliculogenesis-related molecules was investigated by quantitative real-time RT-PCR in cultured rat GCs at concentrations of resveratrol between 10 and 100 μM. After 24 h culture, resveratrol significantly increased mRNA levels of LH-R, steroidogenic acute regulatory protein (StAR), and P450arom at 100 μM (Figure 4B-D), while FSH receptor (FSH-R) mRNA levels remained unchanged (Figure 4A). Western blot analysis was also performed to confirm the result of quantitative real time RT-PCR and resveratrol-dependent stimulation of StAR, LH-R, and P450arom protein was confirmed (Figure 4E). To investigate the possibility that resveratrol promote steroidgenesis, serum concentration of P4 was evaluated and it has been revealed that resveratrol exhibited 3-fold enhancement of hormonal secretion at 48 h of culture (Figure 4F).

## Discussion

Recently, resveratrol has been the focus of many *in vitro *and *in vivo *studies because of its pleiotropic biological activities [1-9]. However, the studies of resveratrol in ovarian physiology are limited. Resveratrol has been reported to exert estrogenic effects, increasing uterine and ovarian wet weight [23]. It is a phytoestrogen known to bind equally to estrogen receptors α and β [24], and structurally similar to synthetic estrogens, such as DES and 17β-estradiol benzoate [25]. In contrast to its hyperproliferative effects, resveratrol promoted apoptosis in rat ovarian theca-interstitial cells [12], and its analogues inhibited swine GC growth [13]. It was also reported that resveratrol inhibited the proliferation of a wide variety of human cancer cell lines through the induction of S-phase cell cycle arrest and apoptosis [5]. In the present study, we demonstrated that resveratrol exerted a dose-dependent inhibition of cell viability on rat GCs. This effect appeared not to be due to the induction of apoptosis, which was different from the previous findings in rat ovarian theca-interstitial cells [12]. Then we studied whether this resveratrol-induced decrease of cellular viability may lead to the differentiation of GCs.

Sirtuins are a conserved family of NAD^+^-dependent class III histone deacetylases involved in a number of cellular processes including gene silencing at telomere and mating loci, DNA repair, recombination, and aging [8,10,11]. Recent studies have established that SIRT1 plays an important role in the regulation of cell fate and stress response in mammalian cells, and promotes cell survival by inhibiting apoptosis or cellular senescence induced by stresses including DNA damage [11]. Indeed, resveratrol administration and accompanying activation of SIRT1 has improved health and survival of mice on a high-calorie diet by ameliorating insulin resistance [9]. Here we demonstrated the expression of SIRT1 in human GCs by immunohistochemical and Western blot analysis, and the expression of its mRNA in rat GCs by RT-PCR. To our knowledge, this is the first report that SIRT1 is expressed in the ovarian follicular cells. It is also interesting that resveratrol treatment caused an increase in SIRT1 mRNA levels as well as the stimulation of the deacetylating function of SIRT1. Similar to this result, the mouse experimental model of dextran sodium sulfate-induced colitis was associated with a decrease in SIRT1 gene expression and resveratrol treatment significantly reversed the expression of SIRT1 [26]. However, it should be noted that all actions of resveratrol are not related to the activation of SIRT1 because resveratrol is an indirect activator of SIRT1 and has been shown to activate the expression and activity of nicotinamide phosphoribosyltransferase and AMP-activated protein kinase (AMPK) [27-30].

In our study, resveratrol increased the expression of P450arom and luteinization-related molecules, such as LH-R and StAR, in rat GCs, suggesting a possibility that resveratrol may promote steroidogenesis and luteinization, a process of terminal differentiation of GCs, in the ovary. In fact, P4 secretion was increased after resveratrol treatment. These findings are consistent with the previous study using HL60 promyelocytic cell line [31]. Since mRNA levels of LH-R in the human corpus luteum were reported to be about 7 times higher than those in preovulatory follicles [32], LH-R has been thought to be a key factor in the ability of GC to undergo luteinization. In the human and other primates, StAR is also essential for the development and maintenance of the corpus luteum. StAR is known to govern the rate-limiting step in steroidogenesis, which is the translocation of cholesterol from the outer to the inner mitochondrial membrane [33]. The process of luteinization is associated with up-regulation of StAR in luteinized granulosa cells. Considering the fact that StAR is not highly expressed in GCs of preovulatory follicles [34], our data may implicate a role of SIRT1 and its activator in promoting luteinization of the ovary. Following ovulation, GCs undergo luteinization and form the corpus luteum which secretes P4. Secretion of P4 is indispensable to cause secretory transformation of the endometrium so that implantation can occur. Before the placenta takes over P4 production, P4 produced by the corpus luteum provides the necessary support to early pregnancy. A defect in the corpus luteum function is associated with implantation failure and miscarriage [35]. Here we showed a new insight that the resveratrol treatment may serve, at least in part, as luteal support. Physiological roles of resveratrol in the ovary should be further determined because another possible beneficial effect on ovarian physiology is reported. Resveratrol is known as a pure aryl hydrocarbon receptor antagonist with no agonistic activity. Polycyclic aromatic hydrocarbons are environmental toxicants found in cigarette smoke, and stimulate aryl hydrocarbon receptor. Polycyclic aromatic hydrocarbons have detrimental effect on ovarian reserve via inducing Harakiri, and resveratrol may exert its rescuing effect by inhibiting Harakiri expression [36]. However, in view of the significant difference in the ovarian physiology between humans and rodents, our data should be interpreted with caution and the present observations should be verified using human GCs.

## Conclusions

We have demonstrated that resveratrol plays a key role in the activation of luteinization, the terminal differentiation of GCs, and exerts its effects by stimulating the expression of SIRT1, StAR, LH-R, and P450arom in GCs. As a result of these effects, we found that resveratrol promoted P4 secretion. These results suggest that the stimulation of SIRT1 by resveratrol would be potentially beneficial in the treatment of luteal phase deficiency. Several chemical compounds are known to affect the SIRT1 activities, and SIRT1 stimulators are currently extensively investigated for the treatment of diabetes. We hypothesize that these drugs might have a role in ovarian physiology by affecting SIRT1, but further studies are necessary to confirm the physiological implication of SIRT1 in the ovary.

## Abbreviations

DES: Diethylstilbestrol; DMEM: Dulbecco's Modified Eagle Medium; FBS: Fetal bovine serum; FSH-R: Follicle stimulating hormone receptor; GC: Granulosa cell; LH-R: Luteinizing hormone receptor; P4: Progesterone; P450arom: P450 aromatase; RT-PCR: Reverse transcript-polymerase chain reaction; StAR: Steroidogenic acute regulatory protein.

## Competing interests

The authors declare that they have no competing interests.

## Authors' contributions

YM carried out all of the experiments. AS, MH, HH, YM, and KS participated in the immunohistochemistry, real time PCR, Western blot, and hormonal quantification. OY helped to collect and purify rat GCs. SK, MH, and HO helped to collect human luteinized GCs from follicular aspirates. OW-H has been involved in acquisition of data, drafting the manuscript, and revising it critically for important intellectual content. KO, SN, and TY have made substantial contributions to conception and design, analysis and interpretation of data. KT and YT have given final approval of the version to be submitted. All authors read and approved the final manuscript.
